# Temporal Analysis of Patient-Centered Sentiment in Clinical Notes for Patients With Mental Health Conditions: Retrospective Cohort Study

**DOI:** 10.2196/96771

**Published:** 2026-09-14

**Authors:** AbdulRahman Morsy, Carson J Peters, Leslie Miller, Aya Zirikly

**Affiliations:** 1 Department of Computer Science School of Engineering and Applied Sciences George Washington University Washington, DC United States; 2 Center for Excellence in Public Health Leadership Kennedy Krieger Institute Baltimore, MD United States; 3 Department of Psychiatry and Behavioral Sciences School of Medicine Johns Hopkins University Baltimore, MD United States; 4 Center for Speech and Language Processing Whiting School of Engineering Johns Hopkins University Baltimore, MD United States

**Keywords:** clinical notes, electronic health records, large language models, mental health, MIMIC, sentiment analysis, temporal sentiment analysis

## Abstract

**Background:**

Clinical notes offer rich, longitudinal insights into patient health trajectories. However, existing clinical sentiment analysis primarily evaluates overall note tone rather than the patient’s distinct perspective. This gap is particularly critical in mental health care, where patient-perspective sentiment closely correlates with severe clinical outcomes, including mortality.

**Objective:**

This study aimed to characterize temporal sentiment trajectories and changes in mental health clinical notes. We are specifically interested in the problem from the patient perspective, as this can differ from the overall and the provider-perspective sentiment. Furthermore, we examine its association with postdischarge mortality to check if there is any correlation. Using large language models (LLMs) and lexicon-based methods, we compared and highlighted the strengths and weaknesses of both approaches.

**Methods:**

We conducted a retrospective analysis of 16,447 clinical notes from 6,382 patients from the Medical Information Mart for Intensive Care IV (MIMIC-IV) database, focusing on the Brief Hospital Course and Discharge Instructions sections for patients with *ICD-10* (*International Classification of Diseases*, *Tenth Revision*) mental health diagnoses. Sentiment was labeled from 3 perspectives (patient, physician, and general) using 2 LLMs (DeepSeek-7B and Mistral-7B) and compared to lexicon-based tools (ClinSent-lexicon, TextBlob, and VADER [Valence Aware Dictionary and sEntiment Reasoner]). Temporal trends were quantified at the patient level using Kendall τ. Associations between sentiment patterns and mortality were assessed using independent-samples (Welch) t tests. Model performance was evaluated on a manually annotated subset (n=165) using precision, recall, and *F*_1_-score.

**Results:**

Temporal sentiment trajectories showed substantial directional change among patients with multiple hospital admissions, with greater fluctuations in Discharge Instructions compared to Brief Hospital Course notes. Patient-perspective sentiment was more balanced, while physician and general perspectives were predominantly neutral. LLMs showed better alignment with patient-centered annotations than lexicon-based methods. Discharge-note sentiment trajectories were significantly more negative among patients who died within 30-90 days of discharge than among survivors across both LLMs.

**Conclusions:**

Temporal sentiment analysis revealed section-dependent patterns in clinical narratives that partially reflected patient-perceived experience. LLM-based approaches improved alignment with patient-centered sentiment, although overall performance remained limited. These findings underscore the need for larger, more robust datasets and modeling strategies for clinical sentiment analysis.

## Introduction

### Overview

Sentiment analysis, the task of identifying affective polarity, emotional tone, and subjective attitudes in text, has been widely applied in domains such as social media, product reviews, public health surveillance, and health care settings. In the medical domain, sentiment carries additional importance because it may reflect clinically meaningful signals related to patient well-being, disease progression, quality of care, pain management, and stay duration [1-3]. Additionally, sentiment in clinical text differs fundamentally from general-domain sentiment. Rather than expressing opinions or preferences, polarity in clinical notes often reflects patient condition, prognosis, or perceived health status, making it more complex and context-dependent [4,5].

Clinical notes, including progress notes, visit notes, and discharge summaries, provide a rich longitudinal record of patient care. These notes contain detailed descriptions of symptoms, treatments, and responses over time. Prior work in clinical natural language processing (NLP) extensively leveraged such data for tasks including named entity recognition, medical coding, and outcome prediction. Sentiment analysis of clinical notes has also received growing attention, including lexicon-based [6,7] and machine learning approaches [8,9], which is also underscored by recent scoping reviews of the field [1,4]. However, patient-centered sentiment analysis, examining how clinical documentation may be emotionally perceived by patients themselves, rather than sentiment in clinical text more broadly, remains relatively less studied, with gaps in methodological approaches, validation, model interoperability, and robustness of datasets [1,4,10]. Existing studies have largely relied on lexicon-based approaches or traditional machine learning methods, which struggle to capture contextual meaning and domain-specific language [4,6]. General-purpose sentiment tools such as VADER (Valence Aware Dictionary and Sentiment Reasoner) and TextBlob are frequently used for baseline analysis. To better account for the unique linguistic nuances of health care documentation, more recent developments have introduced dedicated clinical sentiment tools such as ClinSent, which offers 2 separate scoring methods: a consensus-based clinical keyword lexicon and a supervised deep learning classifier [7,8].

Transformer-based models, including BERT, BioBERT, nBERT, and ClinicalBERT, have substantially improved performance across a wide range of clinical NLP tasks, particularly in named entity recognition, relation extraction, and clinical text classification [2,9,11]. These models have achieved state-of-the-art results on multiple biomedical and clinical text mining benchmarks. Despite these advances, their application has largely focused on structured prediction tasks rather than sentiment analysis.

More recently, large language models (LLMs) have demonstrated strong capabilities in clinical reasoning and medical text understanding and education [11,12]. However, their use for sentiment analysis in clinical notes remains underexplored. This gap is particularly important when sentiment is framed from the patient perspective, where the objective is to capture how patients might perceive and emotionally interpret their own clinical notes.

Another key limitation in the literature is the lack of temporal analysis. Most studies treat clinical notes as independent observations, ignoring the longitudinal nature of patient records and limiting the depth of the contextual information [4,13]. Temporal sentiment analysis, which examines how sentiment evolves across notes for the same patient, has the potential to reveal meaningful trajectories related to disease progression and treatment response. Despite its importance, this aspect has received limited attention in clinical NLP research.

### Objectives and Research Questions

In this study, we introduced a patient-centered framework for temporal sentiment analysis of clinical notes. Our study addressed gaps in the literature by (1) modeling sentiment as a longitudinal signal across patient records, (2) comparing sentiment interpretations from patient, physician, and general perspectives, and (3) exploring whether temporal sentiment trajectories are associated with clinical outcomes. We defined patient-perspective sentiment as the anticipated emotional impact that reading a clinical note may have on the patient.

Specifically, we investigated the following research questions:

- RQ1: How do LLM-based methods perform relative to lexicon-based approaches in detecting patient-perspective sentiment in clinical notes?
- RQ2: How do patient-perceived sentiment trajectories evolve over time in mental health clinical notes?
- RQ3: How does patient-centered sentiment compare with physician and general annotations, and how does annotator background affect agreement?
- RQ4: Can temporal sentiment trajectories, captured via LLMs or lexicon-based methods, reveal clinically relevant signals, such as associations with postdischarge mortality?

### Contributions

This study makes the following contributions:

1. Longitudinal patient-centered analysis: a novel analysis of sentiment in mental health clinical notes is provided, moving beyond static, note-level assessments to capture the evolution of patient-perceived states over time.
2. Multiperspective sentiment mapping: sentiment from patient, physician, and general annotator perspectives is systematically compared, identifying how clinical background significantly influences annotation agreement.
3. LLM vs lexicon exploration: LLMs (DeepSeek-7B and Mistral-7B) are explored against traditional clinical and general lexicons (ClinSent-lexicon, TextBlob, and VADER), demonstrating superior alignment with true patient-perceived sentiment for LLMs.
4. Clinical outcome correlation: temporal sentiment trajectories are examined in relation to postdischarge mortality, motivated by the observation that most observed deaths in this cohort occurred after discharge rather than during hospitalization. Discharge-note sentiment trajectories were more negative among patients who died within 30-90 days of discharge than among survivors, an association that remained significant after correction across the 3 temporal metrics evaluated within each stratum and was most apparent among patients with major depressive disorder (F32), the largest diagnostic subgroup in this cohort.

By addressing both temporal dynamics and perspective-specific interpretations, this work provides a more clinically meaningful understanding of affective signals in electronic health records and lays the foundation for future applications of patient-perspective LLMs in clinical decision support.

## Methods

### Data Sources

#### Overview

We conducted a retrospective study using the Medical Information Mart for Intensive Care IV (MIMIC-IV) database, which contains deidentified electronic health records of patients admitted to critical care units [13]. Access was obtained through PhysioNet, and as the data are fully deidentified, the study was exempt from institutional review board (IRB) approval.

We focused on the Brief Hospital Course (BHC) and Discharge Instructions (Discharge) sections of clinical notes to capture patient-centered and clinician-interpreted language relevant to mental health. The selection of these sections was based on prior work, which identifies these sections as particularly informative for capturing patient-related behavior and summarizing the clinical trajectory [14]. The BHC section contains narrative summaries of hospitalization events, assessments, and treatments, while the Discharge section details guidance provided to patients upon discharge. We have included a synthetic example of a snippet of clinical notes in the Multimedia Appendix 1.

#### Cohort Selection

Given the focus on mental health in this study, our selective cohort of patients mapped to the corresponding 4 major psychiatric diagnostic categories with the following *ICD-10* (*International Classification of Diseases*, *Tenth Revision*) codes: major depressive disorder (F32 and F33), bipolar disorder (F25.0, F30, and F31), eating disorders (F50), and schizophrenia (F20), consistent with prior work [15]. Only patients with at least 2 clinical notes with valid time stamps were included, allowing longitudinal sentiment analyses. For patients with multiple diagnoses, all relevant *ICD-10* codes were retained.

#### Cohort Statistics

Filtering for relevant notes yielded 6382 patients from an initial 9457, contributing 16,447 notes in total. Of these, 1162 patients had multiple hospital admissions, contributing 6003 of those notes, and formed the basis for the temporal sentiment analysis.

### Data Preprocessing

Notes were cleaned to remove empty entries, duplicates after text normalization (lowercasing, whitespace collapsing, and punctuation removal), very short content, and those without valid time stamps. Only the targeted sections were extracted and ordered chronologically to construct longitudinal sentiment trajectories.

### Sentiment Analysis

Sentiment was assessed using LLMs (DeepSeek-7B and Mistral) prompted from 3 perspectives: general, patient, and physician. The general perspective captured the overall sentiment of the note based on the described clinical events and outcomes; the patient perspective focused on the emotional impact the note might have on a patient reading it, independent of medical correctness, aiming to capture the emotional burden, uncertainty, and perceived severity that patients may experience when engaging with their clinical documentation; and the physician perspective evaluated the clinical trajectory and medical implications from a provider’s viewpoint. Each note was classified into 1 of 3 labels (positive, neutral, or negative). The exact prompt templates used for each perspective are provided in Multimedia Appendix 2. Lexicon-based tools (TextBlob, VADER, and ClinSent-lexicon) were included as baselines.

Human annotation was performed on a subset of 165 notes, with 1 clinical expert and 1 nonclinical annotator. The clinical expert has a strong background in patient engagement and an understanding of clinical diagnoses from a patient-informed understanding. Annotators evaluated each clinical note from the patient’s perspective along 2 sentiment dimensions. The first dimension, Emotional Impact Only (Excluding Medical Facts), assessed the immediate emotional response elicited by the note’s tone and language while explicitly disregarding the clinical content and medical implications. The second dimension, Emotional and Medical Impact (Including Medical Facts), evaluated sentiment by considering both the patient’s emotional reaction and the potential real-world implications of the documented medical information on the patient’s health status and treatment trajectory.

This design ensured patient-centered perspectives while evaluating the effect of clinical expertise. On the 165-note subset, interannotator agreement was substantial in both settings: when medical facts were included, agreement reached a Cohen κ of 0.763 with 87.27% (144/165) agreement, while excluding medical facts yielded a Cohen κ of 0.71 with 86.06% (142/165) agreement.

The gold-standard annotations are imbalanced, with neutral (99/165, 60%), negative (59/165, 35.8%), and positive (7/165, 4.2%). A majority-class classifier that always predicts neutral achieves 60.0% accuracy. The expected chance agreement under the empirical label distribution is 0.49, which serves as the baseline for Cohen κ. These baselines indicate that model performance should be interpreted in the context of strong class imbalance and nontrivial chance agreement.

### Temporal Modeling

For each patient, sentiment labels were ordered chronologically based on note time stamps to construct longitudinal trajectories. Temporal changes in sentiment were characterized using multiple complementary measures.

First, a first-last comparison was used to classify trajectories as improving, worsening, or stable by comparing the sentiment of the earliest and latest notes, with stability defined as no change between the first and last sentiment labels.

Second, mean consecutive change was computed as the average difference between adjacent sentiment labels, where values near zero indicated stable sentiment with minimal short-term fluctuation, positive values indicated improvement, and negative values indicated worsening.

Third, Kendall τ correlation was used to quantify monotonic trends in sentiment over time by measuring how consistently sentiment moves in one direction across successive notes. Intuitively, it reflects whether later notes tend to be more positive or more negative than earlier ones. τ values near zero indicate no consistent directional trend (stable or fluctuating sentiment), positive values indicate an overall improvement in sentiment over time, and negative values indicate an overall worsening.

Fourth, a half-mean comparison was performed by comparing the average sentiment of the first half of notes with that of the second half, with similar averages indicating stable sentiment, higher second-half averages indicating improvement, and lower second-half averages indicating worsening.

In addition, dominant sentiment and class proportions (positive, neutral, and negative) were computed to summarize overall sentiment tendencies. Sentiment labels were mapped to numeric values (positive=1, neutral=0, and negative=–1) to enable quantitative analysis and aggregation at the patient level.

These complementary measures captured both global trends and local variations in sentiment trajectories. The selection of nonparametric, interpretable measures rather than parametric longitudinal models, such as linear regression or mixed-effects models, was motivated by our primary aim of characterizing and describing sentiment trajectory patterns across a heterogeneous set of irregularly spaced clinical notes, rather than fitting predictive longitudinal models to individual patient trajectories.

The models selected are DeepSeek-7B and Mistral-7B, each evaluated under the 3 prompting perspectives (general, patient, and physician), TextBlob, VADER, and ClinSent-lexicon. The rationale for model selection encompassed various reasons, with particular strengths of each model, such as speed, accuracy, complexity or simplicity, and discernment of tone.

### Outcome Measure

To evaluate temporal sentiment and model performance, we defined multiple outcome measures at both the note and patient levels.

1. Model performance: on the manually annotated subset, we computed macroaveraged precision, recall, and *F*_1_-scores for each sentiment detection method. These metrics quantify the agreement between automated predictions and patient-perspective human annotations. The choice of macroaveraged metrics is due to class imbalance in the gold annotations (neutral: 99/165, 60%, negative: 59/165, 35.8%, and positive: 7/165, 4.2%), since macro *F*_1_ assigns equal weights to all classes, and therefore avoids inflation from the majority class, which would otherwise dominate weighted metrics. This is particularly important in our setting, where correct identification of minority sentiment classes is clinically meaningful.
2. Sentiment class distribution: for each model and perspective, we calculated the proportion of notes assigned to positive, neutral, and negative classes. This measure characterizes overall polarity tendencies across the cohort.
3. Patient-level temporal trends: longitudinal sentiment trajectories were summarized per patient as proportions of notes exhibiting improvement, stability, or worsening. Trajectories were quantified using the first-last comparison, half-mean comparison, mean consecutive change, and Kendall τ metrics.
4. Section- and diagnosis-specific trends: temporal trends were computed separately for BHC and Discharge Instructions sections, and for each *ICD-10* mental health diagnosis code to detect section-specific and diagnosis-specific patterns.
5. Mortality associations: associations between patient-level sentiment trend scores and postdischarge mortality were assessed using independent-samples (Welch) *t* tests comparing deceased and surviving patients, both overall and stratified by *ICD-10* diagnostic category.

### Statistical Analysis

Descriptive statistics were used to summarize cohort characteristics and sentiment distributions across sections and *ICD-10*-coded diagnoses. For each patient, sentiment trajectories were quantified using multiple metrics derived from the time-ordered notes, including first-to-last trend, mean consecutive change, half-mean change, Kendall τ, dominant sentiment, positive/negative ratios, and net sentiment.

Comparisons of sentiment patterns were performed across *ICD-10* codes and between sentiment extraction methods (LLM vs lexicon-based tools). To explore potential clinical relevance, associations between sentiment trajectories and postdischarge mortality were examined for both LLM- and lexicon-derived sentiment using descriptive and trajectory-level metrics.

All statistical tests were 2-sided, with a significance threshold of *P*<.05. CIs were reported where appropriate.

For mortality association analyses, a Bonferroni correction (α=.05/3≈.017) was applied across the 3 temporal trend metrics tested within each analysis stratum (first-last, Kendall τ, and half-mean).

### Data Exclusion

Notes were excluded if they were empty, duplicated, or lacked valid time stamps. Patients with fewer than 2 eligible notes were excluded, as temporal trajectories could not be constructed. Additional filtering was applied to remove notes with insufficient textual content for reliable sentiment classification.

### Ethical Considerations

This study was a retrospective secondary analysis using the MIMIC-IV database [11]. The data were originally collected and deidentified by the Massachusetts Institute of Technology Laboratory for Computational Physiology in accordance with the HIPAA (Health Insurance Portability and Accountability Act) Safe Harbor standards.

The researchers obtained access to the database via PhysioNet after completing the required training. Because the dataset is fully deidentified and publicly available for credentialed researchers, this study was deemed exempt from IRB oversight. In strict compliance with the data use agreement, no attempts were made to reidentify any individuals or contact patients. All analysis was performed on secure, password-protected systems to ensure data integrity.

## Results

### Model Evaluation Against Human Annotations

Table 1 reports model performance against the consensus subset of patient-perspective human annotations, where both annotators independently agreed (n=144, 87.27% of the full 165-note set), including 95% bootstrap CIs. Sentiment detection aligned most closely when LLMs were prompted from the patient perspective. General and physician perspectives yielded lower *F*_1_-scores, while lexicon-based tools consistently underperformed LLM-based approaches. The CIs largely preserved the same ranking, supporting the robustness of the findings despite overlap due to the relatively small evaluation set (n=144). Across both model families, patient-perspective prompting remained the most human-aligned configuration, suggesting that prompt framing strongly influences sentiment interpretation. Because LLaMA-3-8B and BioClinical ModernBERT (zero-shot) underperformed relative to the DeepSeek-7B and Mistral-7B configurations across all 3 perspectives, these 2 models were excluded from the subsequent temporal trend and mortality association analyses. Expanded results for both models, together with a supervised fine-tuning experiment for BioClinical ModernBERT, are reported in Multimedia Appendix 3.

**Table 1:** Performance of large language model and lexicon-based sentiment tools against patient-perspective annotations with 95% CIs.

Comparison	Precision (95% CI)	Recall (95% CI)	*F*_1_-score (95% CI)
Mistral-7B patient	0.418 (0.351-0.486)	0.363 (0.310-0.417)	0.369 (0.311-0.429)
Mistral-7B general	0.407 (0.330-0.484)	0.350 (0.243-0.503)	0.335 (0.266-0.406)
DeepSeek-7B patient	0.379 (0.308-0.449)	0.510 (0.338-0.620)	0.333 (0.259-0.406)
Mistral-7B physician	0.404 (0.297-0.513)	0.371 (0.276-0.521)	0.316 (0.248-0.395)
DeepSeek-7B physician	0.402 (0.232-0.580)	0.381 (0.293-0.541)	0.312 (0.228-0.427)
DeepSeek-7B general	0.402 (0.171-0.550)	0.340 (0.333-0.354)	0.255 (0.226-0.291)
Llama-3-8B general	0.199 (0.160-0.249)	0.342 (0.255-0.496)	0.245 (0.201-0.309)
Llama-3-8B physician	0.191 (0.164-0.218)	0.334 (0.333-0.333)	0.243 (0.220-0.263)
Llama-3-8B patient	0.190 (0.162-0.217)	0.325 (0.312-0.333)	0.239 (0.216-0.260)
BioClinical ModernBERT physician	0.251 (0.190-0.308)	0.263 (0.208-0.317)	0.236 (0.182-0.291)
BioClinical ModernBERT general	0.248 (0.188-0.306)	0.258 (0.203-0.313)	0.231 (0.179-0.285)
TextBlob	0.208 (0.169-0.249)	0.404 (0.209-0.556)	0.231 (0.185-0.280)
BioClinical ModernBERT patient	0.241 (0.181-0.297)	0.253 (0.198-0.305)	0.227 (0.175-0.279)
VADER^a^	0.400 (0.160-0.560)	0.364 (0.210-0.562)	0.226 (0.180-0.276)
ClinSent-lexicon	0.270 (0.198-0.340)	0.456 (0.415-0.503)	0.208 (0.147-0.273)

^a^VADER: Valence Aware Dictionary and Sentiment Reasoner.

### Sentiment Class Distributions

Table 2 summarizes the distribution of positive, neutral, and negative classes across models and perspectives. Patient-focused prompts yielded more balanced distributions, while general and physician perspectives were heavily skewed toward neutral. Lexicon-based tools displayed inconsistent distributions, highlighting how model choice and perspective shape outputs.

**Table 2:** Distribution of sentiment classes across models and perspectives.

Sentiment column	Positive, n (%)	Neutral, n (%)	Negative, n (%)
DeepSeek-7B general	12 (0.07)	16330 (99.29)	105 (0.64)
DeepSeek-7B patient	7101 (43.18)	7597 (46.19)	1749 (10.63)
DeepSeek-7B physician	739 (4.49)	15339 (93.26)	369 (2.24)
Mistral-7B general	3709 (22.55)	10730 (65.24)	2008 (12.21)
Mistral-7B patient	1608 (9.78)	11668 (70.94)	3171 (19.28)
Mistral-7B physician	1926 (11.71)	13726 (83.46)	795 (4.83)
TextBlob	6384 (38.82)	9859 (59.94)	204 (1.24)
VADER^a^	8810 (53.57)	158 (0.96)	7479 (45.47)
ClinSent-lexicon	9784 (59.49)	2948 (17.92)	3715 (22.59)

^a^VADER: Valence Aware Dictionary and Sentiment Reasoner.

The distributions emphasize that patient-perspective prompting captures nuanced sentiment, whereas general and physician prompts favor neutral responses, and lexicon-based tools vary considerably. This highlights the importance of both model and prompt role in clinical sentiment analysis.

### Temporal Trends Across Perspectives

#### Overview

Patient-level sentiment trajectories revealed substantial differences across models and perspectives. Using general LLM prompts, most patients exhibited stable sentiment, with minimal instances of improvement or worsening. In contrast, patient-perspective prompts captured more dynamic trajectories, with a notable proportion of patients improving or worsening over time. Physician-perspective prompts generally reflected stability, but still identified some patients with improving or worsening trajectories.

Lexicon-based tools demonstrated variable patterns: VADER and TextBlob captured moderate numbers of improving patients, whereas ClinSent-lexicon identified a higher proportion of worsening trajectories, particularly under half-mean analysis.

Comparisons between DeepSeek-7B and Mistral-7B LLMs highlighted that patient-perspective prompting consistently increased sensitivity to temporal changes, whereas general or physician perspectives tended to underrepresent improvement and deterioration. Half-mean metrics and Kendall trends provided complementary assessments of longitudinal change, confirming the overall patterns observed with first vs last note and mean-difference analyses.

Table 3 summarizes patient-level sentiment trends comparing DeepSeek-7B and Mistral-7B, restricted to the 1162 patients with multiple hospital admissions. Overall, DeepSeek-7B detects a higher proportion of patients with improving sentiment (655/1162, 56.4% by Kendall trend) compared to Mistral-7B (459/1162, 39.5%). Both models detect substantial temporal change across this multiadmission cohort, with DeepSeek-7B skewing more heavily toward improvement and Mistral-7B showing a more even distribution across improving, stable, and worsening trajectories. These differences highlight that DeepSeek-7B and Mistral-7B differ in their sensitivity to temporal variation in patient-perceived sentiment across notes.

**Table 3:** Patient-level sentiment trends: DeepSeek-7B vs Mistral.

Method—metric	Improving, n (%)	Stable, n (%)	Worsening, n (%)
DeepSeek-7B—First-Last	574 (49.4)	355 (30.55)	233 (20.05)
DeepSeek-7B—Half Mean	427 (36.75)	371 (31.93)	364 (31.33)
DeepSeek-7B—Kendall	655 (56.37)	100 (8.61)	407 (35.03)
Mistral-7B—First-Last	339 (29.17)	655 (56.37)	168 (14.46)
Mistral-7B—Half Mean	370 (31.84)	457 (39.33)	335 (28.83)
Mistral-7B—Kendall	459 (39.50)	340 (29.26)	363 (31.24)

#### Dominant Sentiment

Across all models, neutral sentiment dominated in general and physician-perspective prompts, which is expected given the inherently objective nature of clinical notes, while patient-perspective models captured a more balanced distribution of positive and negative perceptions. Lexicon-based tools showed inconsistent distributions, with some overestimating positive sentiment and others identifying higher negative sentiment prevalence, as shown in Table 2. The stronger neutrality in physician-perspective prompts likely reflects the standardized and detached style of medical documentation, where clarity and objectivity are prioritized. In contrast, patient-perspective prompting introduces subjective interpretation and emotional context, producing a more balanced distribution of positive and negative sentiment. This suggests that sentiment in clinical text is highly dependent on interpretive perspective, not just surface-level wording.

### Key Observations

#### Overview

Patient-perspective LLM prompts captured more dynamic sentiment trajectories, identifying a higher proportion of patients exhibiting either improvement or worsening over time, which suggests closer alignment with subjective patient experiences. In contrast, physician- and general-perspective prompts, as well as certain lexicon-based tools, predominantly indicated stable sentiment across patients. Across all temporal metrics, half-mean trends and Kendall τ correlations corroborated the patterns observed in first-last and mean-difference trends, highlighting the robustness and consistency of the temporal sentiment trajectories.

#### Patient-Perspective Sentiment Trends: BHC vs Discharge Instructions

We compared patient-perspective sentiment trends captured by DeepSeek-7B and Mistral-7B across BHC and discharge sections, using 3 temporal trend metrics: first-last, Kendall τ, and half-mean. Differences between models were tested using the Stuart-Maxwell test of marginal homogeneity, a paired-data generalization of the McNemar test to more than 2 categories, with 95% CIs for each between-model difference obtained via 2000 paired bootstrap resamples. Discharge notes were generally more dynamic than BHC notes across both models, with higher proportions of patients showing improving or worsening sentiment. Mistral-7B tended to report slightly more stability than DeepSeek-7B in both sections, whereas DeepSeek-7B captured more pronounced sentiment change, particularly at discharge. The Kendall metric remained the most stable across both sections, while half-mean trends were most sensitive to intermediate variation.

Table 4 shows that discharge notes were more variable than BHC notes across both models, indicating that patient-perceived sentiment may shift substantially over the course of hospitalization. Paired significance testing confirmed that the between-model differences were statistically significant across all 3 metrics in discharge notes (Stuart-Maxwell *P*<.01), but only for the first-last metric in BHC notes (*P*=.046); differences by the Kendall and half-mean metrics in BHC notes were not statistically significant (*P*=.41 and *P*=.21, respectively). These results indicate that DeepSeek-7B and Mistral-7B diverge most in their sensitivity to sentiment change at discharge, while their patient-perspective classifications during the hospital course are largely concordant except when measured by the more change-sensitive first-last metric.

**Table 4:** Patient-perspective sentiment trends (% of patients) for DeepSeek-7B and Mistral-7B across Brief Hospital Course (BHC) and discharge notes. Stability, improvement, and worsening are aggregated per trend metric.

Section, metric, and model			Stable, n (%)			Improving, n (%)		Worsening, n (%)		*P* values	
**BHC**											
	**First-last**										.046
		DeepSeek-7B	687 (59.2)		237 (20.4)		237 (20.4)				
		Mistral-7B	745 (64.2)		204 (17.6)		212 (18.3)				
	**Mean difference**										.046
		DeepSeek-7B	687 (59.2)		237 (20.4)		237 (20.4)				
		Mistral-7B	745 (64.2)		204 (17.6)		212 (18.3)				
	**Kendall**										.41
		DeepSeek-7B	979 (84.3)		86 (7.4)		96 (8.3)				
		Mistral-7B	995 (85.7)		83 (7.1)		83 (7.1)				
	**Half mean**										.21
		DeepSeek-7B	632 (54.4)		277 (23.9)		252 (21.7)				
		Mistral-7B	671 (57.8)		247 (21.3)		243 (20.9)				
**Discharge instructions**											
	**First-last**										.001
		DeepSeek-7B	571 (49.1)		304 (26.2)		287 (24.7)				
		Mistral-7B	654 (56.3)		265 (22.8)		243 (20.9)				
	**Mean difference**										.001
		DeepSeek-7B	571 (49.1)		304 (26.2)		287 (24.7)				
		Mistral-7B	654 (56.3)		265 (22.8)		243 (20.9)				
	**Kendall**										.01
		DeepSeek-7B	937 (80.6)		103 (8.9)		122 (10.5)				
		Mistral-7B	972 (83.6)		85 (7.3)		105 (9.0)				
	**Half mean**										<.001
		DeepSeek-7B	494 (42.5)		339 (29.2)		329 (28.3)				
		Mistral-7B	590 (50.8)		291 (25.0)		281 (24.2)				

#### Patient-Perspective Sentiment Trends: Per ICD-10 Code

Patient-perspective sentiment trajectories were examined across *ICD-10* prefixes to determine whether different diagnostic categories exhibited distinct longitudinal patterns. Table 5 summarizes the proportion of patients exhibiting stable, improving, or worsening sentiment trajectories in BHC and discharge sections of MIMIC-IV clinical notes, with corresponding sample sizes and 95% CIs.

**Table 5:** Patient-perspective sentiment trends per ICD-10 (International Classification of Diseases, Tenth Revision) prefix (% of patients) in Brief Hospital Course (BHC) and discharge notes.

*ICD-10* prefix and section			Stable, % (95% CI)	Improving, % (95% CI)		Worsening, % (95% CI)
**F20 (n=52)**						
	BHC	53.8 (40.4-67.3)		26.9 (15.4-38.5)	19.2 (9.6-30.8)	
	Discharge	34.6 (23.1-48.1)		38.5 (25.0-51.9)	26.9 (15.4-38.5)	
**F25.0 (n=12)**						
	BHC	83.4 (58.3-100.0)		8.3 (0.0-25.0)	8.3 (0.0-25.0)	
	Discharge	16.7 (0.0-41.7)		50.0 (25.0-75.0)	33.3 (8.3-58.3)	
**F31 (n=172)**						
	BHC	57.6 (50.0-65.1)		22.1 (16.3-28.5)	20.3 (14.5-26.2)	
	Discharge	52.3 (44.8-59.9)		25.0 (18.6-32.0)	22.7 (16.8-29.7)	
**F32 (n=1014)**						
	BHC	57.6 (54.4-60.5)		21.1 (18.7-23.8)	21.3 (18.9-23.7)	
	Discharge	51.4 (48.3-54.4)		23.9 (21.2-26.5)	24.8 (22.1-27.3)	
**F33 (n=64)**						
	BHC	53.1 (40.6-65.6)		18.8 (9.4-28.1)	28.1 (17.2-39.1)	
	Discharge	48.4 (37.5-60.9)		26.6 (15.6-37.5)	25.0 (15.6-35.9)	
**F50 (n=23)**						
	BHC	36.4 (18.2-54.5)		27.3 (9.1-45.5)	36.4 (18.2-59.1)	
	Discharge	34.8 (13.0-56.5)		30.4 (13.0-52.2)	34.8 (17.4-56.5)	

Overall, psychotic disorders (F20 and F25) were characterized by predominantly stable sentiment in both BHC and discharge notes, reflecting consistent patient-perceived experiences over time. In contrast, mood and affective disorders (F30-F33) demonstrated more dynamic trajectories, with higher proportions of patients exhibiting either improvement or worsening sentiment, particularly in discharge notes. Eating disorders (F50) displayed heterogeneous patterns, suggesting variable recovery trajectories and treatment responses across patients. Across most *ICD-10* categories, discharge notes captured more pronounced temporal changes than BHC notes, highlighting the sensitivity of patient-perspective sentiment analysis to detect clinically meaningful shifts. Comparisons between DeepSeek-7B and Mistral-7B indicated that DeepSeek-7B identified a greater proportion of patients with improving sentiment, whereas Mistral-7B classified more patients as stable, particularly in psychotic disorders.

These results demonstrate that patient-perspective sentiment analysis not only reflects expected patterns of stability and variability across diagnostic categories, but also captures nuanced temporal dynamics that may be overlooked by general or physician-perspective prompts.

#### Alignment With Expected Clinical Trajectories

To evaluate the clinical validity of patient-perspective sentiment trends, we compared the observed trajectories with established knowledge for different *ICD-10* categories. Psychotic disorders (F20 and F25) typically exhibit stable symptomatology over time, which aligns with the predominance of stable sentiment observed in both BHC and discharge notes [16,17]. Mood and affective disorders (F30-F33) often show variable courses influenced by treatment response and psychosocial factors, consistent with the higher proportions of patients showing improvement or worsening sentiment in our analysis [18]. Eating disorders (F50) are characterized by heterogeneous recovery patterns, which is reflected in the mixed trajectories detected across both sections of the clinical notes [19].

These comparisons indicate that patient-perspective sentiment analysis captures clinically coherent temporal dynamics and reflects expected patterns across diagnostic categories. The results further highlight that patient-perspective prompting detects nuanced changes more effectively than general or physician-perspective prompts, supporting its use for clinically meaningful sentiment analysis in discharge notes.

### Mortality Prediction

Among patients included in the temporal sentiment analysis, 7.6% (88/1162) died in hospital, and 27.5% (320/1162) died after discharge; that is, 78.4% (320/408) of known deaths in this analysis cohort occurred after discharge. Discharge note sentiment trend scores were more negative among patients who died within 30-90 days of discharge than among survivors. A Bonferroni correction (α=.05/3≈.017) was applied across the 3 temporal trend metrics tested within each analysis stratum (first-last, Kendall τ, and half-mean); these associations remained significant after this correction, for both DeepSeek-7B and Mistral-7B. At 30 days postdischarge, mean first-last sentiment trend scores were negative among deceased patients and positive among survivors for Mistral-7B (mean –0.224 vs +0.060); DeepSeek-7B showed the same directional pattern and reached significance at the 90-day window (mean –0.135 vs +0.065). No comparable association was observed for BHC notes. Full results for the complete set are provided in Multimedia Appendix 4.

Stratified by *ICD-10* category, this association was most apparent in F32 (major depressive disorder), where both models independently showed more negative discharge sentiment trends among patients who died within 30-90 days of discharge (DeepSeek-7B: mean –0.227 deceased vs +0.057 survivors at 30 days; Mistral-7B: mean –0.278 deceased vs +0.045 survivors at 30 days). The F32 subgroup showed the most consistent association. However, F32 was also the largest diagnostic subgroup, providing greater statistical power than smaller *ICD-10*categories. Therefore, this finding should be interpreted as exploratory, rather than as evidence of a diagnosis-specific effect. Smaller subgroups (F31, F33, F50, F20, and F25.0) did not show comparable associations; a small number of per-*ICD-10* comparisons with very few deceased patients were excluded as unreliable and are flagged accordingly in Multimedia Appendix 4.

## Discussion

### Principal Results

This study presents an exploratory analysis of temporal sentiment patterns in clinical notes, with a focus on patient-centered interpretations. Overall, sentiment trajectories among patients with multiple hospital admissions demonstrated predominantly directional and moderate-to-strong monotonic associations over time, with median absolute Kendall τ values of approximately 0.41 for both models. However, direction and magnitude varied considerably across patients, with some trajectories improving, others worsening, and a minority showing no consistent trend. This variability was particularly evident in Discharge Instructions, where sentiment shifts may reflect changes in treatment plans, evolving patient conditions, or differences in how clinical information is communicated at transitional points of care.

A central finding is that sentiment is highly dependent on perspective. Patient-perspective prompting produced substantially different sentiment distributions compared to physician- and general-perspective prompts, which were overwhelmingly neutral. This indicates that sentiment in clinical text is not an intrinsic property of the text alone, but is strongly influenced by the interpretive lens applied. Prompt design, therefore, plays a critical role in shaping sentiment outputs and may have a larger impact than model choice alone. To ensure reproducibility, all prompts used for general, patient, and physician perspectives are provided in Multimedia Appendix 2. Detection and precision of clinical diagnoses continue to be further developed, for example, among mental health challenges and in clinical settings. Emerging research suggests the promise of diagnostic modeling for early detection, based on patient perspective and clinical practice.

LLM-based approaches demonstrated improved alignment with patient-perspective annotations compared to lexicon-based methods. However, overall performance remained modest (*F*_1_-score < 0.5), underscoring the difficulty of the task. Unlike general-domain sentiment, clinical sentiment is often implicit, context-dependent, and tied to descriptions of patient condition rather than explicit opinions, making both annotation and automated detection inherently challenging.

Discharge section sentiment trajectories were associated with postdischarge mortality, with patients who died within 30-90 days of discharge exhibiting more negative sentiment trends than survivors. This association remained significant after Bonferroni correction across the 3 temporal trend metrics evaluated within each analysis stratum and was observed across both DeepSeek-7B and Mistral-7B, whereas no comparable association was found for in-hospital mortality. These findings should be interpreted cautiously, however, as the analysis was exploratory, correction for multiple comparisons was limited to temporal metrics within each analysis stratum, the association was most apparent in the largest diagnostic subgroup: major depressive disorder (F32), and the retrospective design supports association rather than causation or clinical prediction.

Finally, differences between LLMs (eg, DeepSeek-7B vs Mistral) suggest variability in sensitivity to temporal changes, with DeepSeek-7B skewing more heavily toward detecting improvement while Mistral-7B showed a more even distribution across trend categories. These differences likely reflect both model characteristics and prompt design, reinforcing the importance of careful methodological choices in clinical sentiment analysis.

### Comparison With Prior Work

Prior research in clinical sentiment analysis has largely treated clinical notes as independent observations and has relied on lexicon-based approaches or traditional machine learning methods. In contrast, this study adopts a longitudinal perspective, modeling sentiment as a temporal signal across patient records [4]. Our findings suggest that temporal variation captures meaningful aspects of patient experience, with moderate-to-strong monotonic associations present in a majority of patients with multiple hospital admissions.

Recent advances in transformer-based models and LLMs have improved performance across a range of clinical NLP tasks, but their application to sentiment analysis remains limited. Consistent with prior work, lexicon-based methods in this study showed inconsistent and often biased sentiment distributions [4]. Assessment of public health sentiment analysis continues to be refined and scaled for public opinion, as demonstrated by Rasool et al [20], who assessed the accuracy of public health tweets using the Hybrid Sentiment Method for Correlation. LLMs demonstrated relatively better alignment with patient-perspective interpretations, although performance remained constrained by the complexity of the task.

A key contribution of this work is the explicit comparison of sentiment across multiple perspectives (patient, physician, and general). The results reveal substantial divergence between these perspectives, supporting the view that sentiment in clinical text is inherently subjective and context-dependent. This extends prior literature by demonstrating that capturing patient-perceived sentiment requires not only improved models, but also appropriate framing of the task through perspective-aware prompting. Emerging literature exploring emotional patterns and emotional alignment using nBERT provides context for improving diagnostic accuracy and personalized care, to be integrated into clinical practice, and in this case, mental health [21].

Additionally, this study contributes to emerging work on temporal analysis in clinical NLP by showing that even weak or nonmonotonic sentiment signals may still provide insights when analyzed longitudinally, particularly in sections such as Discharge Instructions.

### Limitations

This study has several limitations. First, the human-annotated evaluation set was relatively small (n=165), which may limit the robustness of model performance comparisons. Expanding the annotated dataset would provide more reliable evaluation; however, manual annotation of clinical notes is resource-intensive and requires both linguistic and clinical expertise, which constrains scalability.

Second, the postdischarge mortality analysis was based on all-cause mortality and could not distinguish cause-specific outcomes (eg, suicide). In addition, this exploratory analysis was not adjusted for all potential confounders, such as illness severity or number of admissions. Therefore, the observed associations should be interpreted cautiously and require validation in larger cohorts using more comprehensive statistical modeling.

Third, our human annotation framework relies on third-party proxy labels rather than direct feedback from patients, which is unattainable with a retrospective electronic health record dataset such as MIMIC. Consequently, this approach is subject to the inherent limitations of manual sentiment labeling, including annotator bias and subjectivity. Although we used both clinical and nonclinical annotators to provide complementary perspectives and minimize individual bias, these text-derived interpretations reflect the patient’s emotional valence as decoded by our annotators from documented narratives. These interpretations, as a proxy framework, may not fully represent the diversity of patient experiences or perceptions, which could influence the alignment between annotated sentiment and real-world patient understanding. To mitigate this, we enforced multiple rounds of calibration and a strict adjudication protocol to ensure the high consistency and reliability of our labels.

Finally, sentiment in clinical text is inherently difficult to define and measure. Unlike general-domain sentiment, it is rarely expressed explicitly and is often intertwined with descriptions of symptoms, prognosis, and treatment. This makes both annotation and automated detection challenging and introduces ambiguity in interpretation.

### Future Work

This work provides an introduction to sentiment polarity (positive, neutral, and negative) within mental health care contexts, particularly among complex care patients and vulnerable patient groups. Future work should focus on building on emotion-specific labeling (ie, anxious, hopeful, and frustrated), which provides further clinically actionable and novel insights informing clinical and public health practice [22].

Our analysis focused on specific note sections (Brief Hospital Course and Discharge Instructions) and selected *ICD-10* mental health diagnoses. Further studies are encouraged to assess generalization to other clinical domains, specialties, or note types.

Future work should also explore mixed-effects or other parametric longitudinal models to formally capture individual patient trajectories and between-patient variability, complementing the descriptive nonparametric measures used here.

### Conclusion

This study introduces a patient-centered framework for temporal sentiment analysis of clinical notes and provides preliminary insights into how sentiment evolves across patient records. The findings suggest that sentiment in clinical notes is highly dependent on perspective, with sentiment trajectories among patients with multiple hospital admissions showing moderate-to-strong monotonic associations over time. Discharge section sentiment trajectories showed an association with postdischarge mortality, particularly among patients with major depressive disorder, suggesting that patient-perspective sentiment captured in discharge notes may carry signal relevant to outcomes beyond the hospital stay; this association is exploratory, noncausal, and based on a single retrospective cohort, and should be validated in larger, prospective cohorts with cause-specific mortality data and confounder-adjusted modeling before informing clinical use.

While LLM-based approaches improve alignment with patient-perspective sentiment compared to lexicon-based methods, overall performance remains limited, highlighting the complexity of the task. Future work should focus on expanding annotated datasets, improving modeling strategies, and incorporating richer clinical context to better capture patient-perceived sentiment. More robust statistical analyses and larger cohorts will be essential to determine whether temporal sentiment signals can be reliably used in clinical decision support or outcome prediction.

## Appendix

Multimedia Appendix 1 Synthetic and clarification of perspectives.

Multimedia Appendix 2 Models and prompts.

Multimedia Appendix 3 Extended model comparison and fine-tuning experiment.

Multimedia Appendix 4 Discharge sentiment trajectories and postdischarge mortality.

## Abbreviations

BHC: Brief Hospital Course
HIPAA: Health Insurance Portability and Accountability Act
ICD-10: International Classification of Diseases, Tenth Revision
IRB: institutional review board
LLM: large language model
MIMIC: Medical Information Mart for Intensive Care
NLP: natural language processing
VADER: Valence Aware Dictionary and sEntiment Reasoner

## Data Availability

The data used in this study were obtained from the Medical Information Mart for Intensive Care (MIMIC-IV) database available on PhysioNet. While these datasets are publicly accessible, access requires completion of the relevant Collaborative Institutional Training Initiative trainings and acceptance of the associated data use agreement.
