# Supplementary material for: Temporal Analysis of Patient-Centered Sentiment in Clinical Notes for Patients With Mental Health Conditions: Retrospective Cohort Study
Source: J Med Internet Res. 2026 Sep 14;28:e96771. doi: 10.2196/96771 (PMC13620431; doi:10.2196/96771)
Supplement: Supplementary file 1 [file jmir_v28i1e96771_app1.pdf]

Multimedia Appendix 2: Example of Perspective-Dependent Sentiment Interpretation

To illustrate the distinction between general, patient-centered, and physician-centered sentiment analysis, we provide a synthetic example of a discharge instructions section modeled after clinical notes.

Both the synthetic example and analysis are provided by ChatGPT-5.5.
To comply with the privacy policy of MIMIC dataset, no examples were provided to the model.

Synthetic Discharge Instructions Example

DISCHARGE INSTRUCTIONS:
You were admitted for worsening shortness of breath and chest pain and were found to have acute decompensated heart failure. During your hospitalization, you responded well to IV diuretics with improvement in breathing and reduction in leg swelling. Your oxygen requirements resolved, and your weight decreased by 6 kg.

Please continue taking furosemide, lisinopril, and metoprolol as prescribed. Follow a strict low-sodium diet and restrict fluid intake to less than 1.5 liters daily. Monitor your weight every morning and contact your doctor if you gain more than 2 pounds in one day or 5 pounds in one week.

Your echocardiogram showed a reduced ejection fraction of 25%, which means your heart function remains severely weakened. This condition is chronic and requires close outpatient follow-up. You are at increased risk for recurrent hospitalization if medications or diet are not followed carefully.

Follow up with cardiology within 7 days. Return to the emergency department immediately for worsening shortness of breath, chest pain, dizziness, or fainting.

Sentiment Interpretation Across Perspectives

General Perspective (Neutral). Under a general sentiment framework, this note would likely be interpreted as neutral because it contains both positive and negative elements. While the patient improved during hospitalization and was deemed stable for discharge, the note also describes a serious chronic condition with substantial future risk, creating an overall balanced sentiment.

Patient-Centered Perspective (Negative). From the patient's perspective, the sentiment would likely be negative, as emotionally salient phrases such as *"heart function remains severely weakened," "chronic,"* and *"increased risk for recurrent hospitalization"* may provoke anxiety, fear, or uncertainty. Despite the improvement, the emotional burden of the prognosis may dominate the interpretation.

Physician-Centered Perspective (Positive). From the physician's perspective, the sentiment would likely be positive because the focus is on clinical trajectory and treatment response. Relative to admission, the patient demonstrated improvement in symptoms, resolution of oxygen requirements, and successful stabilization, supporting discharge readiness despite the chronic underlying condition.

This example demonstrates how the same clinical text may yield different sentiment labels depending on the interpretive framework. While physician-centered sentiment reflects clinical trajectory and general sentiment captures overall informational balance, patient-centered sentiment captures emotional burden and perceived prognosis. This distinction motivates our focus on patient-centered sentiment analysis as a complementary lens for understanding clinical documentation.
