# Supplementary material for: Temporal Analysis of Patient-Centered Sentiment in Clinical Notes for Patients With Mental Health Conditions: Retrospective Cohort Study
Source: J Med Internet Res. 2026 Sep 14;28:e96771. doi: 10.2196/96771 (PMC13620431; doi:10.2196/96771)
Supplement: Supplementary file 2 [file jmir_v28i1e96771_app2.pdf]

Multimedia Appendix 1: Models and Prompts

Model Specifications and Configurations

The following 7-billion parameter models were utilized for sentiment classification tasks. To ensure reproducibility and eliminate stochastic variance, both models were implemented using greedy decoding (do_sample=False).

- DeepSeek-7B (deepseek-ai/deepseek-llm-7b-chat): A chat-optimized large language model. It was configured with a maximum input sequence length of 1024 tokens and a 3-token generation limit to ensure a single-label response (Positive, Neutral, or Negative).
- Mistral-7B (mistralai/Mistral-7B-Instruct-v0.2): An instruction-tuned LLM optimized for explicit prompt adherence. It utilized the [INST] instruction format with a maximum input sequence length of 2048 tokens and a 3-token generation limit.

Perspective-Specific Prompt Templates

General Perspective Prompt (PROMPT_GENERAL_LLM)

```
You are analyzing a clinical note. Determine the overall sentiment conveyed in
the note based purely on what it describes.

Choose ONLY ONE label: positive, neutral, or negative.

Definitions:
- positive: The note describes improvement, recovery, relief, or beneficial
outcomes.
- neutral: The note describes stable status, routine findings, or no meaningful
change.
- negative: The note describes worsening condition, complications, or
concerning findings.

Respond with ONLY the label.

Clinical note:
{text}

Sentiment:
```

Patient Perspective Prompt (PROMPT_PATIENT_LLM)

```
You are the patient reading your own clinical note. Focus ONLY on how this
information would make you feel emotionally.

Choose ONLY ONE label: positive, neutral, or negative.

Definitions:
- positive: The note would make you feel reassured, hopeful, relieved, or
encouraged.
- neutral: The note would not strongly affect your emotions; it feels routine
or informational.
```

```
- negative: The note would make you feel worried, anxious, discouraged, or
concerned.

Base your decision on emotional impact - not medical correctness.
Respond with ONLY the label.

Clinical note:
{text}

How I feel after reading this note:
```

Physician Perspective Prompt (PROMPT_PHYSICIAN_LLM)

```
You are a physician reviewing this clinical note. Assess the clinical
trajectory and medical implications described.

Choose ONLY ONE label: positive, neutral, or negative.

Definitions:
- positive: The patient is clinically improving, responding well to treatment,
or showing beneficial progress.
- neutral: The patient is clinically stable, with no significant improvement or
deterioration.
- negative: The patient is worsening, experiencing complications, or showing
concerning clinical signs.

Respond with ONLY the label.

Clinical note:
{text}

Physician assessment:
```
