# Supplementary material for: Temporal Analysis of Patient-Centered Sentiment in Clinical Notes for Patients With Mental Health Conditions: Retrospective Cohort Study
Source: J Med Internet Res. 2026 Sep 14;28:e96771. doi: 10.2196/96771 (PMC13620431; doi:10.2196/96771)
Supplement: Supplementary file 3 [file jmir_v28i1e96771_app3.pdf]

Multimedia Appendix 3: Extended Model Comparison and Fine-Tuning Experiment

This appendix has two parts. Section A3.1 shows how LLaMA-3-8B and BioClinical ModernBERT performed against the human annotations, alongside the models already shown in Table 1. Both models performed worse than DeepSeek-7B and Mistral-7B, so they were not carried forward into the temporal trend and mortality analyses in the main text. Section A3.2 reports a separate experiment where we fine-tuned BioClinical ModernBERT on our annotated data and checked whether this improved its performance compared to using it without any training. Section A3.3 included per-label metrics for all models.

A3.1. LLaMA-3-8B and BioClinical ModernBERT Performance

Appendix Table 3a. Performance of LLaMA-3-8B and BioClinical ModernBERT (general, patient, and physician perspectives) against the consensus subset of patient-perspective annotations, with 95 percent bootstrap confidence intervals (N=144).

Model	Precision	Recall	F1
Llama-3-8B general	0.199 (0.160, 0.249)	0.342 (0.255, 0.496)	0.245 (0.201, 0.309)
Llama-3-8B physician	0.190 (0.164, 0.218)	0.333 (0.333, 0.333)	0.242 (0.220, 0.263)
Llama-3-8B patient	0.189 (0.162, 0.217)	0.325 (0.312, 0.333)	0.239 (0.216, 0.260)
BioClinical ModernBERT physician	0.250 (0.190, 0.306)	0.262 (0.208, 0.313)	0.235 (0.182, 0.288)
BioClinical ModernBERT general	0.248 (0.188, 0.305)	0.258 (0.203, 0.312)	0.231 (0.179, 0.283)
BioClinical ModernBERT patient	0.240 (0.181, 0.296)	0.252 (0.198, 0.305)	0.227 (0.175, 0.278)

A3.2. BioClinical ModernBERT Fine-Tuning Experiment

We fine-tuned BioClinical ModernBERT as a sentiment classifier on the full 165-note annotated dataset. The data were split into a 70 percent training set (115 notes) and a 30 percent held-out test set (50 notes). We measured the untrained (zero-shot) model on the test set, then trained for five epochs and measured it again on the same test set.

Table 3b. Gold-standard label distribution in the annotated dataset (N=165).

Sentiment Label	N	Percent of Annotated Set (N=165)
Neutral	99	60.0%
Negative	59	35.8%
Positive	7	4.2%

Table 3c. Performance before and after fine-tuning, on the held-out test set (N=50).

Model	Class	Precision	Recall	F1	N
Zero-Shot (Base)	Negative	0.00	0.00	0.00	18
	Neutral	0.55	0.70	0.62	30
	Positive	0.00	0.00	0.00	2
	Macro Average	0.18	0.23	0.21	50
Fine-Tuned	Negative	0.62	0.72	0.67	18
	Neutral	0.76	0.73	0.75	30
	Positive	0.00	0.00	0.00	2
	Macro Average	0.46	0.49	0.47	50

Table 3d. Overall accuracy and macro F1, before and after fine-tuning.

Model	Accuracy	Macro F1	N
Zero-Shot (Base)	0.42	0.21	50
Fine-Tuned	0.70	0.47	50

A3.3. Per-Class Performance for All Evaluated Models

The main text reports macro-averaged precision, recall, and F1 for each model. Macro averaging treats all three sentiment classes equally, which can hide how a model does on any one class. The table below breaks each model's performance down by class.

Table 3g. Precision, recall, and F1 by sentiment class for all evaluated models against the consensus subset (N=144), sorted by Macro F1 descending.

Model	Neutral			Negative			Positive			Macro F1
	P	R	F1	P	R	F1	P	R	F1	
Mistral-7B patient	0.603	0.756	0.670	0.652	0.333	0.438	0.000	0.000	0.000	0.369
Mistral-7B general	0.581	0.573	0.576	0.613	0.282	0.383	0.027	0.196	0.046	0.335
DeepSeek-7B patient	0.576	0.329	0.417	0.478	0.404	0.435	0.082	0.797	0.146	0.333
Mistral-7B physician	0.586	0.792	0.673	0.579	0.123	0.201	0.047	0.196	0.074	0.316
DeepSeek-7B physician	0.559	0.891	0.686	0.505	0.053	0.095	0.142	0.198	0.155	0.312
DeepSeek-7B general	0.575	1.000	0.729	0.626	0.017	0.034	0.000	0.000	0.000	0.254
Llama-3-8B general	0.545	0.830	0.657	0.000	0.000	0.000	0.052	0.196	0.079	0.245
Llama-3-8B physician	0.571	1.000	0.726	0.000	0.000	0.000	0.000	0.000	0.000	0.242
Llama-3-8B patient	0.568	0.975	0.717	0.000	0.000	0.000	0.000	0.000	0.000	0.239
BioClinical ModernBERT physician	0.434	0.242	0.309	0.315	0.544	0.397	0.000	0.000	0.000	0.235
TextBlob	0.568	0.621	0.592	0.000	0.000	0.000	0.057	0.591	0.102	0.231
BioClinical ModernBERT general	0.432	0.230	0.299	0.312	0.544	0.395	0.000	0.000	0.000	0.231
BioClinical ModernBERT patient	0.413	0.230	0.294	0.308	0.527	0.387	0.000	0.000	0.000	0.227
VADER	0.631	0.012	0.024	0.541	0.686	0.603	0.028	0.394	0.052	0.226
ClinSent-Lexicon	0.552	0.269	0.360	0.189	0.106	0.135	0.070	0.994	0.129	0.208
