# Supplementary material for: Temporal Analysis of Patient-Centered Sentiment in Clinical Notes for Patients With Mental Health Conditions: Retrospective Cohort Study
Source: J Med Internet Res. 2026 Sep 14;28:e96771. doi: 10.2196/96771 (PMC13620431; doi:10.2196/96771)
Supplement: Supplementary file 4 [file jmir_v28i1e96771_app4.pdf]

Multimedia Appendix 4: Discharge Sentiment Trajectories and Post-Discharge Mortality

This appendix supports the "Association Between Discharge Sentiment Trajectories and Post-Discharge Mortality" subsection of the Results. For each combination of section (BHC or discharge), sentiment model (DeepSeek-7B or Mistral-7B), post-discharge mortality outcome (within 30 days or overall), and trend metric (First-Last, Kendall’s tau, or half-mean), we compared the mean trend score between patients who died and patients who survived using a two-sample t-test, applying a Bonferroni correction (n=3) across the three trend metrics within each stratum. This was tested overall and separately within each ICD diagnostic prefix, for 480 combinations in total (60 overall + 420 per-ICD-prefix); 11 met the corrected significance threshold and are listed in Appendix Table 5a below.

Appendix Table 5a. Associations between discharge-note sentiment trends and post-discharge mortality for tests meeting the within-stratum significance threshold.

Stratum	Section	Model	Outcome	Metric	N died	N alive	Mean (died)	Mean (alive)	P value
F32	discharge	Mistral-7B	Post-Discharge <=30d	First-Last	97	917	-0.278	0.045	P<.001
Overall	discharge	Mistral-7B	Post-Discharge <=30d	First-Last	107	1055	-0.224	0.060	P<.001
F32	discharge	Mistral-7B	Post-Discharge <=30d	Half-Mean	97	917	-0.228	0.042	P=.001
Overall	discharge	Mistral-7B	Post-Discharge <=30d	Half-Mean	107	1055	-0.188	0.062	P=.002
Overall	discharge	Mistral-7B	Post-Discharge <=90d	First-Last	208	954	-0.125	0.068	P=.004
F32	discharge	Mistral-7B	Post-Discharge <=90d	First-Last	190	824	-0.142	0.050	P=.007
Overall	discharge	Mistral-7B	Post-Discharge <=90d	Half-Mean	208	954	-0.093	0.068	P=.01
F32	discharge	DeepSeek-7B	Post-Discharge <=90d	First-Last	190	824	-0.168	0.075	P=.01
F32	discharge	Mistral-7B	Post-Discharge <=90d	Half-Mean	190	824	-0.104	0.044	P=.04
F32	discharge	DeepSeek-7B	Post-Discharge <=30d	First-Last	97	917	-0.227	0.057	P=.04
Overall	discharge	DeepSeek-7B	Post-Discharge <=90d	First-Last	208	954	-0.135	0.065	P=.04
